# All-Trans Retinoic Acid Attenuates Inflammation and Insulin Resistance Induced by Adipocyte–Macrophage Coculture

**DOI:** 10.3390/molecules30204111

**Published:** 2025-10-16

**Authors:** Kwang-Rim Baek, Hye-Kyeong Kim

**Affiliations:** Department of Food Science & Nutrition, The Catholic University of Korea, 43 Jibong-ro, Wonmi-gu, Bucheon 14662, Republic of Korea; rimmy@seoultech.ac.kr

**Keywords:** all-trans retinoic acid, adipocyte, macrophage, inflammation, insulin resistance, obesity, NF-κB, GLUT4

## Abstract

Obesity is characterized by chronic low-grade inflammation, largely driven by macrophage infiltration into adipose tissue, which contributes to the development of insulin resistance. All-trans retinoic acid (ATRA), a biologically active metabolite of vitamin A, has demonstrated anti-inflammatory properties. This study examined the effects of ATRA on inflammation and insulin resistance using a coculture model comprising hypertrophied 3T3-L1 adipocytes and RAW264.7 macrophages. Coculture markedly elevated the production of pro-inflammatory mediators—including nitric oxide, monocyte chemoattractant protein-1, tumor necrosis factor-alpha, and interleukin-6—and increased free fatty acid release while suppressing the secretion of anti-inflammatory adiponectin. Treatment with ATRA (0.1, 1, and 10 μM) significantly reversed these coculture-induced alterations (*p* < 0.001). ATRA also inhibited the nuclear translocation of NF-κB and downregulated the expression of retinol-binding protein 4 (RBP4). Moreover, ATRA improved insulin-stimulated glucose uptake in adipocytes rendered insulin-resistant by coculture (*p* < 0.01), an effect associated with the restoration of glucose transporter 4 (GLUT4) and insulin receptor substrate-2 (IRS-2) expression. These findings suggest that ATRA effectively mitigates inflammation and insulin resistance arising from adipocyte–macrophage interactions, highlighting its potential as a therapeutic agent for obesity-related metabolic disorders.

## 1. Introduction

Obesity is strongly associated with chronic low-grade inflammation, a key contributor to the development of insulin resistance [1]. Insulin resistance, defined as the diminished responsiveness of insulin-target tissues to insulin, is the central pathogenic mechanism underlying obesity-associated disorders such as type 2 diabetes mellitus (T2DM), non-alcoholic fatty liver disease (NAFLD), and atherosclerosis [2,3]. Adipose tissue plays a pivotal role in this inflammatory state by releasing pro-inflammatory cytokines and free fatty acids (FFAs), which impair insulin signaling in peripheral organs such as the liver and skeletal muscle [4].

In obese adipose tissue, hypertrophic adipocytes become metabolically dysfunctional, exhibiting elevated basal lipolysis and secreting chemokines such as monocyte chemoattractant protein-1 (MCP-1) [5]. MCP-1 recruits circulating monocytes into adipose tissue, where they differentiate into macrophages and adopt a pro-inflammatory M1 phenotype, characterized by the production of cytokines such as tumor necrosis factor-alpha (TNF-α), interleukin-6 (IL-6), and interleukin-1β (IL-1β) [6,7]. Macrophages and adipocytes engage in bidirectional communication through the secretion of various cytokines and chemokines, amplifying local inflammation and exacerbating insulin resistance [8]. Adipocyte-derived factors, including MCP-1, TNF-α, and saturated fatty acids, activate the Toll-like receptor 4 (TLR4) complex, leading to NF-κB activation in resident macrophages and subsequent upregulation of pro-inflammatory cytokines [9,10]. In turn, macrophage-derived TNF-α acts on adipocytes in a paracrine manner, shifting adipokine secretion toward a pro-inflammatory profile and promoting lipolysis by disrupting insulin signaling [10]. Targeting this inflammatory cross-talk between adipocytes and macrophages represents a promising therapeutic strategy for mitigating obesity-induced insulin resistance.

Emerging evidence suggests that vitamin A levels may influence adiposity and metabolic health. Specifically, previous studies have linked vitamin A deficiency to increased adiposity in humans [11,12], suggesting a role for vitamin A metabolites in the regulation of obesity and related metabolic disorders. Notably, treatment with all-trans retinoic acid (ATRA)—a bioactive metabolite of vitamin A—has been shown to reduce body weight and adiposity, while improving glucose tolerance and insulin sensitivity in both lean and obese animal models [13,14,15]. Adipose tissue, a major reservoir of vitamin A, actively participates in retinoid metabolism by converting retinol into active metabolites such as ATRA and retinaldehyde [16]. Adipocytes also express retinoic acid receptors (RARs) and peroxisome proliferator-activated receptor (PPAR β/δ), which mediate ATRA-dependent gene expression [14,17]. Previous studies have demonstrated that ATRA suppresses pro-inflammatory mediator production in lipopolysaccharide (LPS)-stimulated macrophages [18,19] and downregulates chemokine expression in adipocytes [20]. However, its role in modulating the crosstalk between adipocytes and macrophages in the context of obesity remains poorly understood.

The present study aimed to determine whether ATRA exerts antidiabetic effects by disrupting the adipocyte–macrophage interactions within obese adipose tissue. Using a coculture model of hypertrophied 3T3-L1 adipocytes and RAW264.7 macrophages, which mimics the inflamed state of obese adipose tissue, we investigated the direct impact of ATRA on inflammatory mediator production, FFA release, and insulin-stimulated glucose uptake.

## 2. Results

### 2.1. Effect of ATRA on Cell Viability

**Figure 1:**
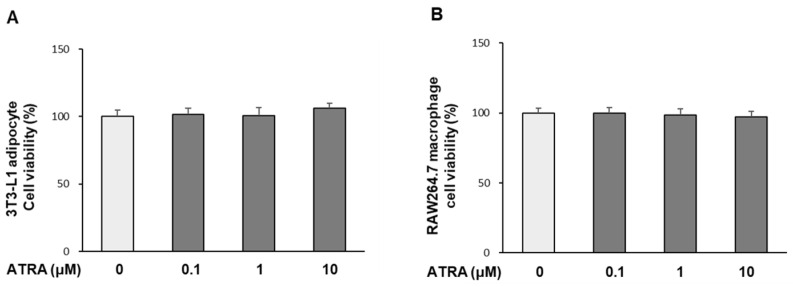
Effect of all-trans retinoic acid (ATRA) on the viability of 3T3-L1 adipocytes and RAW 264.7 macrophages. Cell viability of 3T3-L1 adipocytes (**A**) and RAW 264.7 macrophages (**B**) was assessed using the MTS assay following 24 h exposure to ATRA. Results are expressed as a percentage relative to DMSO vehicle control (defined as 0). Data are presented as mean ± standard deviation (SD) (*n* = 6).

As shown in Figure 1A,B, ATRA exhibited no significant cytotoxicity toward hypertrophied mature adipocytes or RAW 264.7 macrophages at concentrations up to 10 μM, relative to the DMSO vehicle control. Accordingly, all subsequent experiments were conducted using ATRA within this non-cytotoxic range, ensuring functional interaction between adipocytes and macrophages as intact cells.

### 2.2. ATRA Mitigates Inflammatory Responses in Adipocytes–Macrophage Coculture

**Figure 2:**
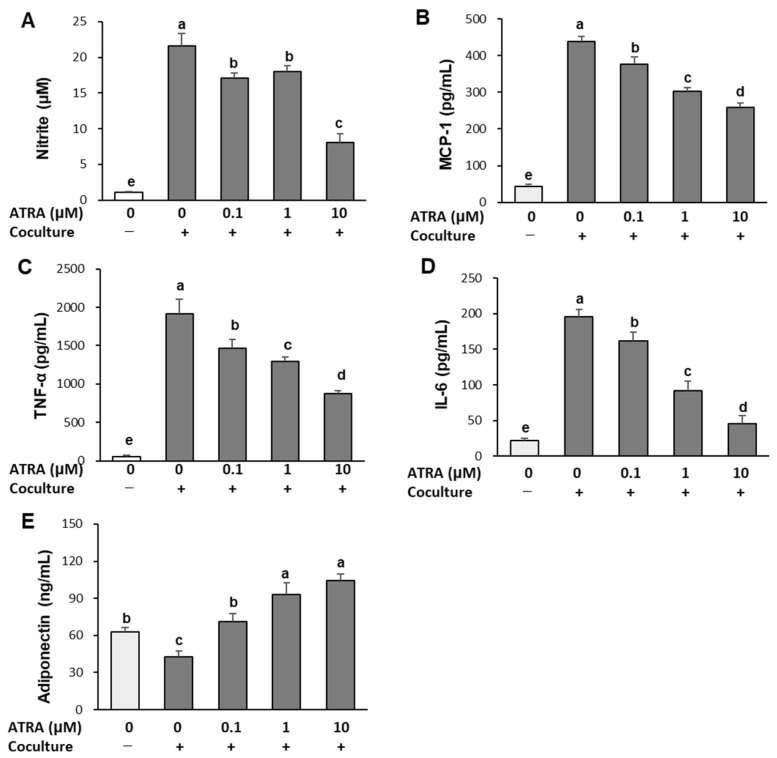
Effect of ATRA on inflammatory responses in cocultured adipocytes and macrophages. Hypertrophied 3T3-L1 adipocytes were cocultured with RAW 264.7 macrophages for 24 h and treated with ATRA for an additional 24 h. The levels of NO (**A**), MCP-1 (**B**), TNF-α (**C**), IL-6 (**D**), and adiponectin (**E**) were measured in the coculture medium. Values are presented as mean ± SD (*n* = 3). Values with different superscript letters are significantly different (*p* < 0.001), as determined by one-way ANOVA followed by Duncan’s multiple range test. +: adipocytes cocultured with macrophages; −: adipocytes and macrophages cultured separately and mixed prior to assay.

Figure 2 illustrates the impact of ATRA on inflammatory responses in cocultured adipocytes and macrophages. In control culture, where adipocytes and macrophages were cultured separately and combined only 1 h prior to the assay, secretion of nitric oxide (NO) and pro-inflammatory cytokines, including TNF-α, MCP-1, and IL-6, was minimal. However, coculture of hypertrophied 3T3-L1 adipocytes with RAW 264.7 macrophages significantly elevated these inflammatory mediators. Notably, ATRA treatment effectively suppressed this coculture-induced increase, even at the lowest concentration of 0.1 μM (*p* < 0.001). Specifically, treatment with ATRA inhibited NO secretion by 63% at 10 μM, and decreased pro-inflammatory cytokine levels in a dose-dependent manner, lowering MCP-1, TNF-α, and IL-6 levels by 39%, 54%, and 76%, respectively, at the same concentration.

Conversely, the production of adiponectin—a key anti-inflammatory adipokine—was markedly reduced in cocultured adipocytes and macrophages compared to separately cultured controls. Treatment with ATRA significantly restored adiponectin secretion, increasing levels by 68%, 113%, and 145% at 0.1, 1, and 10 μM, respectively, compared to cocultured cells (*p* < 0.001). These findings suggest that ATRA exerts potential anti-inflammatory effects in inflamed adipocyte–macrophage environments.

### 2.3. ATRA Reduces Lipolysis in Cocultured Adipocytes

**Figure 3:**
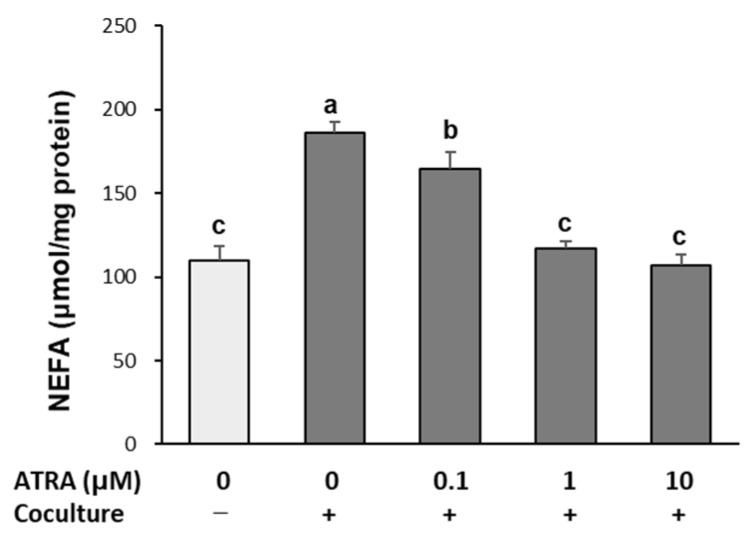
Effect of ATRA on free fatty acid release in cocultured adipocytes and macrophages. Hypertrophied 3T3-L1 adipocytes were cocultured with RAW 264.7 macrophages for 24 h and treated with ATRA for 24 h. Non-esterified fatty acid (NEFA) concentrations in the culture medium were measured using a NEFA assay kit. Data are presented as mean ± SD from three independent experiments. Values with different superscript letters are significantly different (*p* < 0.001), as determined by one-way ANOVA followed by Duncan’s multiple range test. +: adipocytes cocultured with macrophages; −: adipocytes and macrophages cultured separately and mixed prior to assay.

Obesity-associated inflammation promotes adipocyte lipolysis, leading to chronic FFA release and insulin resistance [21]. To assess the effect of ATRA on this process, non-esterified fatty acid (NEFA) levels in the culture medium were measured. As shown in Figure 3, NEFA release increased by 69% in cocultured hypertrophied adipocytes and macrophages compared to control cultures. However, ATRA treatment significantly suppressed this increase (*p* < 0.001), restoring NEFA levels to those observed in control cultures at 1 and 10 μM concentrations.

### 2.4. ATRA Limits NF-κB Activation and RBP4 Expression in Cocultured Adipocytes

**Figure 4:**
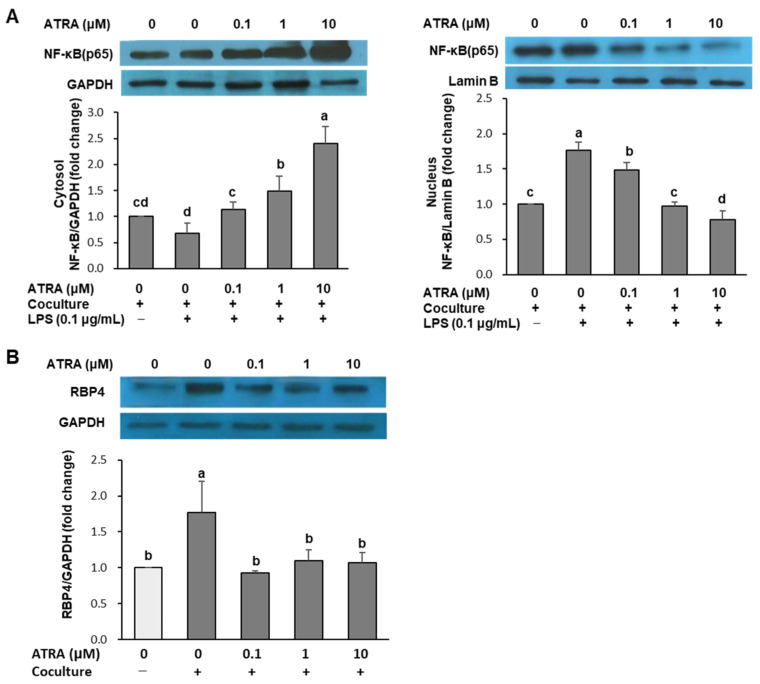
Effect of ATRA on NF-κB signaling (**A**) and retinol-binding protein 4 (RBP4) expression (**B**) in cocultured adipocytes and macrophages. Hypertrophied 3T3-L1 adipocytes were cocultured with RAW 264.7 macrophages for 24 h and treated with ATRA for 24 h. To assess NF-κB activation, cells were stimulated with LPS (0.1 μg/mL) for 30 min, and cytosolic and nuclear levels of the NF-κB p65 subunit were measured. RBP4 expression was assessed via Western blot analysis. Data are presented as mean ± SD from three independent experiments. Values with different superscript letters are significantly different (*p* < 0.001), as determined by one-way ANOVA followed by Duncan’s multiple range test. Coculture +: adipocytes cocultured with macrophages; coculture −: adipocytes and macrophages cultured separately and mixed prior to assay; LPS +: treated with LPS (0.1 μg/mL); LPS −: not treated with LPS.

To elucidate the molecular mechanisms underlying ATRA’s anti-inflammatory effects, NF-κB signaling was examined. NF-κB is a key transcription factor that regulates the expression of pro-inflammatory genes, including inducible nitric oxide synthase (iNOS), TNF-α, IL-6, and MCP-1 [22]. Its activation involves the degradation of cytosolic inhibitor κB proteins (IκB), leading to the dissociation of the IκB−NF-κB complex. This allows NF-κB to translocate into the nucleus and bind to target gene promoters [22]. To assess NF-κB activation, the distribution of p65 subunits in cytoplasmic and nuclear fractions was analyzed. LPS treatment, which induces IκB degradation, resulted in a 35% decrease in cytoplasmic p65 and 1.8-fold increase in nuclear p65 compared to the LPS-untreated coculture control, confirming NF-κB translocation. However, treatment with ATRA at concentrations of 0.1, 1, and 10 μM reduced nuclear translocation of NF-κB p65 by 15%, 44% and 55%, respectively, compared to the LPS-treated coculture control (*p* < 0.001; Figure 4A).

Retinol-binding protein 4 (RBP4), an adipokine responsible for retinol transport in the bloodstream, is known to activate immune cells and stimulate the release of pro-inflammatory cytokines via TLR and c-Jun N-terminal kinase (JNK) pathways [23]. As shown in Figure 4B, RBP4 expression was markedly elevated in cocultures of hypertrophied adipocytes and macrophages, compared to control cultures. Notably, ATRA treatment restored RBP4 expression to levels comparable to the control.

### 2.5. ATRA Enhances Insulin Sensitivity in Cocultured Adipocytes

**Figure 5:**
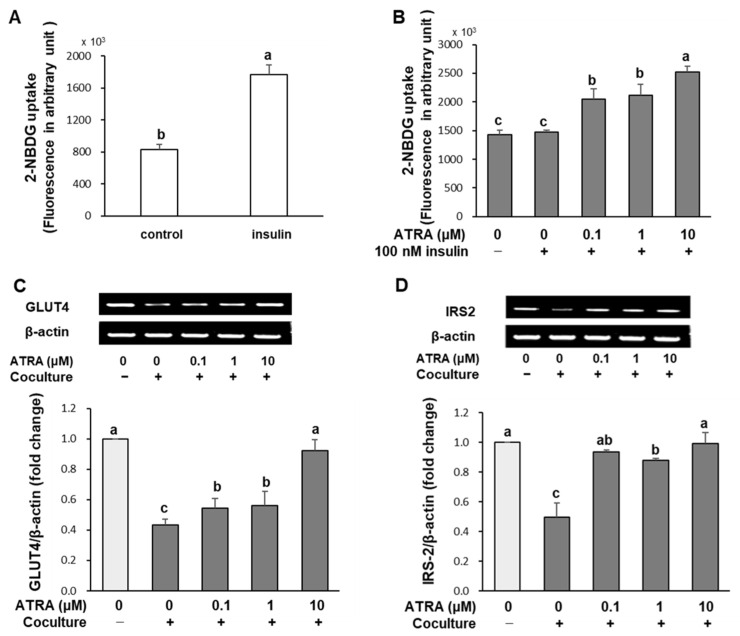
Effect of ATRA on glucose uptake in adipocytes cultured alone (**A**) and cocultured with macrophages (**B**), and mRNA expression of glucose transporter 4 (GLUT4) (**C**) and insulin receptor substrate-2 (IRS-2) (**D**). After coculturing hypertrophied 3T3-L1 adipocytes with RAW 264.7 macrophages for 24 h, the cells were serum-starved and subsequently incubated with ATRA, insulin (100 nM), and the fluorescent glucose derivative 2-NBDG (20 μM) for 4 h. Glucose uptake was quantified by measuring fluorescence intensity (in arbitrary unit). For gene expression analysis, total RNA was extracted from cocultured cells after 24 h of treatment with ATRA, and mRNA levels of target genes were assessed using RT-PCR. Data are presented as mean ± SD (*n* = 6 for glucose uptake; *n* = 3 for RT-PCR). Values with different superscript letters are significantly different (*p* < 0.001), as determined by one-way ANOVA followed by Duncan’s multiple range test. Coculture +: adipocytes cocultured with macrophages; Coculture −: adipocytes not cocultured with macrophages; 100 nM insulin +: treated with insulin; 100 nM insulin −: not treated with insulin.

To evaluate insulin sensitivity, we measured the uptake of 2-[*N*-(7-(nitrobenz-2-oxa-1,3-diazol-4-yl)-amino]-2-deoxylucose (2-NBDG), a fluorescent glucose analog. In 3T3-L1 adipocytes cultured without macrophages, insulin stimulation increased 2-NBDG uptake by more than twofold (Figure 5A). In contrast, cocultured adipocytes showed no significant increase in 2-NBDG uptake upon insulin stimulation, indicating the development of insulin resistance. Treatment with ATRA significantly increased 2-NBDG uptake in cocultured adipocytes and macrophages by 40%, 44%, and 72% at concentrations of 0.1, 1, and 10 μM, respectively, compared to the insulin-treated coculture control (Figure 5B; *p* < 0.01). To elucidate the molecular mechanisms underlying this enhanced glucose uptake, we analyzed the expression of glucose transporter 4 (GLUT4) and insulin receptor substrate 2 (IRS-2). GLUT4 mRNA levels were reduced by 56% in cocultures of hypertrophied adipocytes and macrophages relative to control cultures. ATRA treatment significantly restored GLUT4 expression, reaching levels comparable to the control at 10 μM (Figure 5C; *p* < 0.001). Similarly, IRS-2, a key mediator of insulin-stimulated GLUT4 translocation [24], was downregulated by approximately 50% in cocultured cells relative to control cultures. ATRA treatment effectively reversed this suppression, restoring IRS-2 mRNA expression to near-control levels (Figure 5D; *p* < 0.001).

## 3. Discussion

The interplay between adipocytes and infiltrating macrophages in obese adipose tissue is a critical driver of chronic inflammation and systemic insulin resistance. This study investigated the effects of ATRA on inflammatory responses and adipocyte function in hypertrophied adipocytes cocultured with macrophages and explored the underlying molecular mechanisms.

The direct-contact coculture of hypertrophied 3T3-L1 adipocytes with RAW264.7 macrophages serves as a well-established in vitro model of obese adipose tissue, characterized by a pronounced upregulation of pro-inflammatory cytokines [10,25]. Consistent with previous findings, coculture led to increased secretion of NEFA and inflammatory mediators such as NO, MCP-1, TNF-α, and IL-6 while simultaneously reducing adiponectin levels. ATRA treatment effectively reversed these changes.

The elevated NEFA release observed in coculture likely reflects enhanced lipolysis driven by macrophage-derived TNF-α [10], which contributes to insulin resistance by promoting hepatic glucose production and impairing glucose uptake in skeletal muscle [21]. TNF-α acts as an early inflammatory signal that induces the expression of iNOS, cyclooxygenase-2 (COX-2), and IL-6 via NF-κB activation in 3T3-L1 adipocytes [26]. IL-6 further disrupts insulin signaling by upregulating suppressor of cytokine signaling-3 (SOCS-3), which inhibits phosphorylation of the insulin receptor and IRS proteins [27]. MCP-1 promotes macrophage recruitment, while FFAs amplify macrophage activation and cytokine secretion [9]. ATRA has previously been shown to downregulate chemokine expression in adipocytes and inhibit macrophage migration in TNF-α-conditioned 3T3-L1 media [20]. In this study, ATRA suppressed coculture-induced NO production, which appears to result from reduced iNOS expression through inhibition of NF-κB activation, as previously observed in 3T3-L1 adipocytes [28]. NO is known to promote macrophage polarization toward a pro-inflammatory phenotype [29] and to impair insulin signaling in adipocytes [30]. Therefore, ATRA-mediated suppression of NO production further supports its role in mitigating macrophage-induced insulin resistance. In addition, ATRA restored adiponectin secretion, providing another anti-inflammatory and anti-diabetic mechanism. Adiponectin is known to suppress the production of inflammatory cytokine by inhibiting pro-inflammatory signaling in macrophages [31], and it relieves hyperglycemia and improves insulin sensitivity by acting on muscle and liver [32]. Collectively, these findings suggest that ATRA attenuates basal lipolysis, inflammatory cytokine production, and macrophage recruitment by disrupting adipocyte–macrophage crosstalk, thereby contributing to the reduction in obesity-associated inflammation and insulin resistance.

At the molecular level, NF-κB is a key transcriptional regulator of pro-inflammatory genes in both adipocytes and macrophages [33,34]. In the coculture system, ATRA inhibited LPS-induced nuclear translocation of the NF-κB p65 subunit, consistent with previous reports linking ATRA to reduced chemokine expression and macrophage migration through NF-κB pathway inhibition in adipocytes [20]. In RAW 264.7 macrophages, ATRA also suppressed LPS-induced NF-κB activation and TNF-α production [19]. Taken together, these findings suggest that the anti-inflammatory effects of ATRA in adipocyte–macrophage coculture are mediated, at least in part, through suppression of NF-κB signaling in both cell types.

We also observed that ATRA downregulated RBP4 expression in cocultured adipocytes. RBP4 levels are elevated in obesity [35] and contribute to macrophage activation and pro-inflammatory cytokine release via TLR and JNK pathways [36]. Its suppression by ATRA represents an additional anti-inflammatory mechanism. Moreover, adipocyte-specific RBP4 overexpression in transgenic mice has been shown to induce hepatic steatosis and glucose intolerance while increasing inflammatory markers [37]. Consistently, adipose RBP mRNA expression in obese humans has been positively correlated with inflammatory markers, and RBP treatment stimulated basal lipolysis in human adipocytes [38]. In line with these findings, ATRA treatment in mice has been reported to reduce adipose RBP4 expression and improve glucose tolerance and insulin sensitivity [15,39]. Therefore, suppression of RBP4 may represent a key mechanism by which ATRA attenuates obesity-induced inflammation and insulin resistance.

Impaired insulin-stimulated glucose uptake in adipocytes and muscle is a hallmark of insulin resistance. In our coculture model, adipocytes failed to respond to insulin, whereas treatment with ATRA restored glucose uptake. Normally, insulin binding initiates receptor autophosphorylation and IRS protein activation, triggering PI3K/Akt signaling and promoting GLUT4 expression and translocation [40]. In obesity and T2DM, GLUT4 expression is diminished in adipocytes but remains intact in muscle [41]. IRS proteins are also reduced in insulin-resistant adipocytes—IRS-2 via transcriptional downregulation and IRS-1 through posttranslational mechanism [42]. Consistent with this, we observed reduced GLUT4 and IRS-2 mRNA levels in cocultured adipocytes, which were restored by ATRA. Previous studies have shown that macrophage-derived factors impair insulin signaling in adipocytes by downregulating GLUT4 and IRS proteins [43]. Our data suggest that ATRA mitigates insulin resistance by restoring GLUT4 and IRS-2 expression and dampening macrophage-induced inflammatory signaling.

While previous studies have focused on ATRA’s role as a nuclear receptor ligand and its regulation of adipokines [14,15,39,44], our findings highlight a more direct role in modulating adipocyte–macrophage interactions—a key feature of obesity-related metabolic dysfunction. In this study, coculture of adipocytes and macrophages offers a distinct advantage over monocultures with conditioned medium by enabling direct cell–cell contact and bidirectional communication, allowing both cell types to respond dynamically to each other’s secreted factors as in obese adipose tissue. However, limitations include the use of supraphysiological concentrations of ATRA in vitro and the absence of in vivo validation. Future studies using obese and diabetic animal models are warranted to determine whether ATRA can modulate adipose tissue inflammation and improve systemic insulin sensitivity in vivo.

In conclusion, ATRA suppressed pro-inflammatory mediators, reduced lipolysis, and restored insulin-stimulated glucose uptake in hypertrophied adipocytes cocultured with macrophages. These effects of ATRA were mediated through attenuating inflammatory paracrine interaction between adipocytes and macrophages. Collectively, our findings support the antidiabetic potential of ATRA and underscore its therapeutic potential for obesity-associated insulin resistance.

## 4. Materials and Methods

### 4.1. Materials

ATRA was purchased from Sigma-Aldrich (St. Louis, MO, USA) and dissolved in dimethyl sulfoxide (DMSO; Sigma-Aldrich) to prepare a 100 mM stock solution. The solution was stored in a freezer, protected from light and air. Dulbecco’s modified Eagle medium (DMEM), fetal bovine serum (FBS), bovine calf serum (BCS), and penicillin–streptomycin were obtained from GIBCO (Grand Island, NY, USA). Isobutylmethylxanthine, dexamethasone, and insulin were also sourced from Sigma-Aldrich. The 3-(4,5-dimethylthiazol-2-yl)-5-(3-carboxy-methoxyphenyl)-2-(4-sulfophenyl)-2H-tetrazolium (MTS) assay kit was procured from Promega (Madison, WI, USA). The enzyme-linked immunosorbent assay (ELISA) kits for TNF-α, IL-6, and MCP-1 were purchased from BD Biosciences (San Diego, CA, USA), while the adiponectin ELISA kit and NEFA assay kit were obtained from R&D Systems (Minneapolis, MN, USA) and FUJIFILM Wako Pure Chemical Corporation (Chuo-Ku, Osaka, Japan), respectively. The fluorescent glucose analog 2-NBDG was purchased from Invitrogen (Carlsbad, CA, USA). Nuclear and cytoplasmic extraction reagents were acquired from Thermo Fisher Scientific (Rockford, IL, USA). Primary antibodies specific for NF-κB (p65), glyceraldehyde 3-phosphate dehydrogenase (GAPDH), and lamin B were purchased from Cell Signaling Technology (Danvers, MA, USA), Ab Frontier (Seoul, Republic of Korea), and Santa Cruz Biotechnology (Santa Cruz, CA, USA), respectively. The secondary antibodies included horseradish peroxidase (HRP)-conjugated goat anti-rabbit IgG from Cell Signaling Technology for detecting NF-κB, RBP4, and GAPDH detection, and HRP-conjugated rabbit anti-goat IgG from Abcam (Cambridge, UK) for detecting lamin B.

### 4.2. Cell Culture

3T3-L1 mouse embryo fibroblasts and RAW 264.7 macrophage cells were obtained from the American Type Culture Collection (ATCC; Manassas, VA, USA) and the Korean Cell Line Bank (KCLB, Seoul, Republic of Korea), respectively. Cell culture procedures followed a previously described protocol [45]. Briefly, 3T3-L1 preadipocytes were cultured in DMEM supplemented with 10% BCS until 2 days post-confluence. Differentiation was initiated on day 0 (D0) using induction medium containing 10% FBS, 0.5 µM isobutylmethylxanthine, 1 µM dexamethasone, and 167 nM insulin. After 2 days, cells were maintained in DMEM supplemented with 10% FBS and 167 nM insulin for an additional 2 days, followed by continued culture in DMEM supplemented with 10% FBS. By day 6 (D6), cells exhibited a mature adipocyte phenotype, characterized by visible lipid droplet accumulation, which progressed over time. Adipocytes between day 14 (D14) and day 20 (D20) were used in experiments as hypertrophied 3T3-L1 adipocytes. Adipocytes and macrophages were cocultured using a direct-contact system. RAW 264.7 macrophages, maintained in DMEM supplemented with 10% FBS, were seeded directly onto serum-starved 3T3-L1 adipocytes at a density of 5 × 10^5^ cells/mL. The coculture was incubated in serum-free DMEM for 24 h. As a control culture, 3T3-L1 cells and RAW 264.7 macrophages were cultured separately and combined 1 h prior to assays. Cocultured cells were treated with ATRA at the indicated concentrations or with 0.1% DMSO as a vehicle control.

### 4.3. Cell Viability Assay

To determine non-toxic concentrations of ATRA, cell viability was assessed using the MTS assay. Concentrations of 0.1, 1, and 10 μM were selected based on previous studies in adipocytes and macrophages [20,42], and a 24 h treatment period was chosen to match the conditions of this study and to capture sustained cytokine responses. For the assay, 3T3-L1 cells were seeded in 96-well plates and cultured until day 14 (D14), when they exhibited a hypertrophied mature adipocyte phenotype, and then exposed to media containing ATRA or vehicle alone (0.1% DMSO). Separately, RAW 264.7 macrophages were seeded at a density of 5 × 10^3^ cells/well in 96-well plates, and treated with ATRA or vehicle after 24 h of culture. After 24 h of incubation, the viability of both cells was measured using the MTS assay according to the manufacturer’s instructions. The absorbance of the resulting formazan product was measured at 490 nm using a microplate reader (ELx808; Biotek, Winooski, VT, USA), with absorbance values directly proportional to the number of viable cells.

### 4.4. Measurement of NO and Cytokine Production

Hypertrophied 3T3-L1 adipocytes and RAW 264.7 macrophages were cocultured for 24 h and then treated with ATRA at concentrations of 0.1, 1, and 10 μM. After incubating for another 24 h, cell culture supernatants were collected for analysis. Nitrite accumulation, a stable end product of NO, was quantified using the Griess assay. Briefly, 100 μL of supernatant was mixed with 100 μL of Griess reagent (1% sulfanilamide in 5% phosphoric acid and 0.1% *N*-(1-naphthyl) ethylenediamine in distilled H_2_O). After incubating at 25 °C for 10 min, absorbance at 540 nm was measured using a microplate reader (ELx808; Biotek). Nitrite concentrations were calculated using a standard curve generated with sodium nitrite. The concentrations of MCP-1, TNF-α, IL-6, and adiponectin in the culture supernatants were measured using commercial ELISA kits, following the manufacturer’s instructions.

### 4.5. Lipolysis Assay

Lipolysis was assessed by measuring NEFA released into the medium from adipocytes. Hypertrophied 3T3-L1 cells were cocultured with RAW 264.7 macrophages for 24 h. After washing with phosphate-buffered saline (PBS), cells were incubated for 24 h in Krebs–Ringer bicarbonate buffer (119 mM NaCl, 4.8 mM KCl, 1.28 mM CaCl_2_, 1.2 mM KH_2_PO_4_, 1.2 mM 7H_2_O·MgSO_4_, 0.25 mM NaHCO_3_, 5 mM glucose, 4% bovine serum albumin; pH 7.4) containing ATRA (0.1, 1, and 10 μM). NEFA concentrations in the culture medium were quantified using a commercial assay kit. Protein content from the corresponding cell pellets (approximate 100 mg wet weight) was determined by the Bradford assay, and NEFA release was normalized to protein concentration.

### 4.6. Immunoblot Analysis

Following coculture of hypertrophied 3T3-L1 adipocytes and RAW 264.7 macrophages for 24 h, cells were treated with ATRA (0.1, 1, and 10 μM) for 24 h to evaluate its effect on NF-κB translocation and RBP4 expression. To assess NF-κB activation, LPS (0.1 μg/mL) was added 30 min prior to the end of treatment. Cytoplasmic and nuclear fractions were isolated using commercial extraction reagents according to the manufacturer’s instructions. Separation of fractions was confirmed by immunoblotting for compartment-specific marker proteins: GAPDH for the cytoplasmic fraction and lamin B for the nuclear fraction. Whole cells or subcellular fractions were lysed in ice-cold RIPA buffer (20 mM Tris-HCl, 150 mM NaCl, 1 mM Na_2_EDTA, 1 mM EGTA, 1% NP-40, 1% sodium deoxycholate, 2.5 mM sodium pyrophosphate, 1 mM β-glycerophosphate, 1 mM Na_3_VO_4_, and 1 µg/mL leupeptin; pH 7.4) and incubated on ice for 15 min. Lysates were centrifuged at 10,000× *g* for 10 min at 4 °C, and 30 μg of total protein was resolved using 12% SDS-PAGE and subsequently transferred to nitrocellulose membranes. Membranes were blocked with 5% skim milk in PBS containing 0.1% Tween 20 for 1 h, followed by overnight incubation at 4 °C with primary antibodies against NF-κB, lamin B, and GAPDH (1:1000 dilution) or RBP4 (1:500 dilution). After washing with PBS, membranes were incubated with HRP-conjugated antibodies for 2 h at 25 °C. Immunoblots were visualized by a Western detection kit (Ab Clon, Seoul, Republic of Korea) and quantified using a FluorChem densitometer (FluorChem, Derbyshire, UK) and ImageJ software (version 1.47, National Institutes of Health, Bethesda, MD, USA).

### 4.7. Glucose Uptake Assay

Adipocytes and macrophages were cocultured in 96-well fluorescence plates for 24 h. Cells were then starved in glucose-free PBS for 2 h, followed by treatment with insulin (100 nM), ATRA (0.1, 1, 10 µM), and 2-NBDG (80 µM). Glucose uptake in hypertrophied adipocytes cultured alone was also assessed to compare insulin responsiveness. After 4 h of incubation, cells were washed with cold PBS to remove residual 2-NBDG. The fluorescence retained in the cell monolayers was measured using a fluorescence microplate reader (Synergy Mx; Biotek), at an excitation wavelength of 465 nm and an emission wavelength of 540 nm.

### 4.8. Reverse Transcription Polymerase Chain Reaction (RT-PCR)

After 24 h of ATRA treatment (0.1, 1, 10 µM) in cocultured adipocytes and macrophages, cells were washed with PBS, and total RNA was extracted using an RNA isolation kit (iNtRon Biotechnology, Seongnam, Republic of Korea). RT-PCR was performed using a one-step RT-PCR premix kit (iNtRON Biotechnology, Republic of Korea), following the manufacturer’s instructions. Briefly, 1 μL of total RNA (200 ng), 2 μL of each specific primer (10 pmol), and RNase-free water were added to RT-PCR PreMix tubes to achieve a final reaction volume of 20 μL. PCR amplification was carried out in a thermocycler (Applied Biosystems, Foster City, CA, USA) under the following conditions: initial denaturation at 95 °C for 5 min; 32 cycles of denaturation at 94 °C for 20 s, annealing at 55 °C for 30 s, and extension at 72 °C for 30 s; followed by a final extension at 72 °C for 5 min. The RT-PCR products were resolved on 2% agarose gels containing ethidium bromide and visualized under UV illumination. β-actin was used as a housekeeping gene for normalization. The primer sequences used were as follows: IRS2, 5′-AGCTGGTGGTAGTCATACCC-3′ (forward), 5′-CAGGTTCATATAGTCAGA-3′ (reverse); GLUT4, 5′-CCTAAGACAAGATGCCGTCG-3′ (forward), 5′-CCCTCAGTCATGCTCATCTG-3′ (reverse); β-actin, 5′-ATGGATGACGATATCGC-3′ (forward), 5′-ATGAGGTAGTCTGTCAGGT-3′ (reverse).

### 4.9. Statistical Analysis

Data are presented as mean ± standard deviation (SD). Statistical significance was determined using one-way analysis of variance, followed by Duncan’s multiple range test using SAS software (version 9.4; SAS Institute, Inc., Cary, NC, USA). Statistical significance was set at *p* < 0.05.

## Data Availability

The original contribution presented in this study are included in the article. Further inquiries can be directed to the corresponding author.

## Abbreviations

2-NBDG	2-[*N*-(7-nitrobenz-2-oxa-1,3-diazol-4-yl)-amino]-2-deoxy-d-glucose
ATRA	all-trans retinoic acid
BCS	bovine calf serum
DMEM	Dulbecco’s modified Eagle medium
FBS	fetal bovine serum
FFA	free fatty acids
GLUT4	glucose transporter 4
HRP	horseradish peroxidase
IL	interleukin
IRS	insulin receptor substrate
JNK	c-Jun N-terminal kinase
LPS	lipopolysaccharide
MCP-1	monocyte chemoattractant protein 1
NEFA	non-esterified fatty acid
NF-κB	nuclear factor-κB
NO	nitric oxide
PBS	phosphate-buffered saline
PPAR β/δ	peroxisome proliferator-activated receptor β/δ
RAR	retinoic acid receptor
T2DM	type 2 diabetes
TLR	Toll-like receptor
TNF-α	tumor necrosis factor-alpha
